# Effect of anticoagulant therapy in COVID-19 patients

**DOI:** 10.1007/s12471-021-01574-7

**Published:** 2021-04-16

**Authors:** R. G. Tieleman, F. A. Klok, E. Belfroid, J. Hoogervorst-Schilp, I. Schalkers, C. W. Jansen, H. J. Siebelink

**Affiliations:** 1 Department of Cardiology, Martini Hospital, Groningen, The Netherlands; 2 Department of Internal Medicine, Leiden University Medical Center, Leiden, The Netherlands; 3 Knowledge Institute of Medical Specialists, Utrecht, The Netherlands; 4 Heart Counsel, The Hague, The Netherlands; 5 Netherlands Society of Cardiology, Utrecht, The Netherlands; 6 Department of Cardiology, Leiden University Medical Center, Leiden, The Netherlands

**Keywords:** COVID-19, Anticoagulation, Mortality

## Abstract

**Background:**

In patients hospitalised with COVID-19, an increased incidence of thromboembolic events, such as pulmonary embolism, deep vein thrombosis and stroke, has been reported**.** It is unknown whether anticoagulation can prevent these complications and improve outcome.

**Methods:**

A systematic literature search was performed to answer the question: What is the effect of (prophylactic and therapeutic dose) anticoagulation therapy in COVID-19 patients on cardiovascular and thromboembolic complications and clinical outcome? Relevant outcome measures were mortality (crucial), hospital admission, length of stay, thromboembolic complications (pulmonary embolism, stroke, transient ischaemic attack), need for mechanical ventilation, acute kidney injury and use of renal replacement therapy. Medline and Embase databases were searched with relevant search terms until 17 July 2020. After systematic analysis, eight studies were included. Analysis was stratified for the start of anticoagulation before or during hospital admission.

**Results:**

There was insufficient evidence that therapeutic anticoagulation could improve the outcome in patients hospitalised with COVID-19. None of the studies demonstrated improved mortality, except for one very small Italian study. Furthermore, none of the studies showed a positive effect of anticoagulation on other outcome measures in COVID-19, such as ICU admission, length of hospital stay, thromboembolic complications, need for mechanical ventilation, acute kidney failure or need for renal replacement therapy, except for two studies demonstrating an association between anticoagulation and a lower incidence of pulmonary embolism. However, the level of evidence of all studies varied from ‘low’ to ‘very low’, according to the GRADE methodology.

**Conclusion:**

Analysis of the literature showed that there was insufficient evidence to answer our objective on the effect of anticoagulation on outcome in COVID-19 patients, especially due to the low scientific quality of the described studies. Randomised controlled studies are needed to answer this question.

**Supplementary Information:**

The online version of this article (10.1007/s12471-021-01574-7) contains supplementary material, which is available to authorized users.

## Clinical question

*Should patients with COVID-19 be treated with prophylactic or therapeutic dose anticoagulation to prevent cardiovascular and thromboembolic complications and improve clinical outcome?*

## Introduction

In hospitalised COVID-19 patients an increased incidence of thromboembolic events, pulmonary embolism, deep vein thrombosis and stroke has been reported [1–6]. In an observational study, the use of heparin was associated with lower mortality [7]. It is not known whether anticoagulation in all hospitalised COVID-19 patients improves outcome. Therefore, the guideline committee decided to perform a review of the literature to answer this clinically relevant question.

## Methods

A review of the literature was performed to answer the following question: What is the effect of (prophylactic and therapeutic dose) anticoagulation therapy in COVID-19 patients on cardiovascular and thromboembolic complications and clinical outcome? This question was structured in a PICO format.

**P**opulation: All proven COVID-19 patients (subgroups: home, hospital, intensive care)
**I**ntervention: Use of vitamin K antagonists, low-molecular weight heparin, unfractionated heparin, direct oral anticoagulants
**C**omparison: No use of vitamin K antagonists, low-molecular-weight heparin, unfractionated heparin, direct oral anticoagulants
**O**utcome: Mortality (crucial), hospital admission, intensive care unit (ICU) admission, length of stay, thromboembolic complications (pulmonary embolism, stroke, transient ischaemic attack), need for mechanical ventilation, acute kidney injury, use of renal replacement therapy

### Relevant outcome measures

The relevant outcome measures were mortality (crucial), and hospital admission, length of stay, thromboembolic complications (pulmonary embolism, stroke, transient ischaemic attack), need for mechanical ventilation, acute kidney injury and use of renal replacement therapy (all important).

### Search and select

The databases Medline (via Ovid) and Embase (via Embase.com) were searched with relevant search terms until 17 July 2020. The systematic literature search resulted in 567 hits. See the search strategy for details (Table S1 of the Electronic Supplementary Material). Thirty-two studies were initially selected based on title and abstract screening. After reading the full text, 24 studies were excluded (Table S2 of the Electronic Supplementary Material), and 8 studies were included.

### Summary of literature

The studies were grouped into three groups (A, B, C).

#### Group A: Anticoagulant drug use before hospital admission

Group A described studies in which COVID-19 patients receiving a therapeutic dose of anticoagulants before hospital admission were compared with patients not on anticoagulants or those taking a prophylactic dose.

#### Group B: Prophylactic anticoagulant drug use started at hospital admission

Group B described studies in which COVID-19 patients taking a prophylactic dose of anticoagulants at hospital admission were compared with patients not on anticoagulants.

#### Group C: Mixed or unclear start of anticoagulant therapy

Group C described studies in which COVID-19 patients receiving a therapeutic dose of anticoagulants were compared with patients not on anticoagulants or taking a prophylactic dose. The patients who received a therapeutic dose of anticoagulants formed a mixed group of patients: those who were already taking anticoagulants before hospital admission or received them during hospital admission. In some studies the moment of starting anticoagulants was unclear; these studies were therefore also included in group C.

### Description of studies

#### Group A: Anticoagulant drug use before hospital admission

**Klok** [2] performed an updated analysis of the incidence of the composite outcome of symptomatic acute pulmonary embolism, deep vein thrombosis, ischaemic stroke, myocardial infarction and/or systemic arterial embolism in all COVID-19 patients admitted to the ICUs of two Dutch university hospitals and one Dutch teaching hospital from ICU admission to death, ICU discharge or 22 April 2020, whichever came first. A total of 184 ICU patients were included in the report. The median follow-up duration ranged from 7 to 14 days. All patients received pharmacological thromboprophylaxis and some full-dose anticoagulation. This study was published as a research paper update.

**Tremblay** [8] performed a retrospective analysis of patients with confirmed COVID-19, comparing outcomes among those who were and were not receiving anticoagulants for unrelated indications at the time of COVID-19 diagnosis. To adjust for bias due to non-random allocation of potential covariates among COVID-19 patients, propensity score matching was performed. Propensity scores were calculated using a logistic regression model, adjusting for age, sex, race, Charlson Comorbidity Index and obesity. A total of 3772 patients were included of which 241 received anticoagulants, 672 received antiplatelet therapy and 2859 patients were not taking anticoagulant or antiplatelet therapy at the time of COVID-19 diagnosis. This study was published as a letter to the editor.

**Russo** [9] aimed to evaluate the prevalence of antithrombotic therapies at admission in patients with COVID-19 and the potential association between antithrombotic therapy and acute respiratory distress syndrome (ARDS), as disease clinical presentation, or in-hospital mortality. Altogether 192 consecutive patients with laboratory-confirmed COVID-19 admitted to the emergency department of five Italian hospitals were included in the study. The study population was divided into two groups according to the evidence of ARDS on chest computed tomography at admission. Propensity score weighting adjusted regression analysis was performed to assess the risk of ARDS at admission and death during hospitalisation in patients treated or not with antiplatelet and anticoagulant agents.

**Sivaloganathan** [10] studied the association between pre-admission antiplatelet/anticoagulant use and COVID-19 mortality. The study population comprised those patients with confirmed COVID-19 admitted as inpatients in Brighton and Sussex University Hospitals NHS Trust between the 7 March and 9 April 2020. The case-control group was constructed at a ratio of 1:2 cases to controls, matching for age and sex, selecting from this overall population. A case was defined as being on an anticoagulant or antiplatelet agent before admission. Controls were then selected from the study population with a limited propensity matching by age and sex to two controls who were not taking the medication of interest using a ‘nearest neighbour’ method. Thirty-one cases and 62 controls were included. Data on patients’ drug history were obtained using the Patient Administration System, which was also used to identify patient deaths up to 11 May 2020. This study was published as correspondence.

**Rossi** [11] aimed to assess whether pharmacological cardio-active treatment reduced mortality risk in the setting of COVID-19 interstitial pneumonia. Rossi retrospectively enrolled 70 elderly patients affected by COVID-19 interstitial pneumonia between 25 February and 20 April 2020. All patients were affected by chronic heart disease and they were followed in the divisional outpatient clinic of the Cardiology Unit of the Policlinico of Modena Hospital. The follow-up ended on 5 May 2020. A total of 26/70 patients (37.1%) were treated with direct oral anticoagulants, the underlying indication being pulmonary embolism (*n* = 7, 26.9%), deep vein thrombosis (*n* = 6, 23%) or atrial fibrillation (*n* = 13, 50%). The endpoint of the study was all-cause mortality. A multivariate analysis was performed to assess the relation between age, gender, direct anticoagulant intake and mortality. This study was published as a letter to the editor.

#### Group B: Prophylactic anticoagulant drug use started at hospital admission

**Tang** [7] aimed to validate the usefulness of the Sepsis-Induced Coagulopathy (SIC) score and other coagulation parameters, in screening out patients who can benefit from anticoagulation through retrospective analysis. Consecutive patients with severe COVID-19 admitted to Tongji Hospital of Huazhong University of Science and Technology in Wuhan from 1 January to 13 February 2020 were retrospectively enrolled. Exclusion criteria were a bleeding diathesis, hospital stay <7 days, lack of information about coagulation parameters and medications, and age <18 years. A retrospective review of the characteristics of these patients was performed through the electronic medical record system of the hospital; the medications and outcomes (28-day mortality) were monitored up to 13 March 2020. A total of 449 patients (181 females and 268 males) classified as severe COVID-19 were enrolled into the study. Two hundred and seventy-two (60.6%) patients had one or more chronic underlying diseases, mainly including hypertension (*n* = 177, 39.4%), diabetes (*n* = 93, 20.7%) and heart disease (*n* = 41, 9.1%). Ninety-nine (22.0%) patients received heparin treatment for at least 7 days. A multivariate analysis was performed to assess the relation between anticoagulant intake and mortality.

#### Group C: Mixed or unclear start of anticoagulant therapy

**Paranjpe** [12] assessed the association between administration of in-hospital anticoagulation and survival in a large cohort of hospitalised patients with COVID-19 and described this in a letter to the editor. Between 14 March and 11 April 2020, 2773 patients were hospitalised with laboratory-confirmed COVID-19 within the Mount Sinai Health System in New York City. The authors used a Cox proportional-hazards model to evaluate the effect of systemic therapeutic anticoagulation (including oral, subcutaneous or intravenous forms) on in-hospital mortality. The moment of starting the anticoagulation is not described in the paper. The authors adjusted for age, sex, ethnicity, body mass index, history of hypertension, heart failure, atrial fibrillation, type 2 diabetes, anticoagulation use prior to hospitalisation and admission date. To adjust for differential length of stay and initiation of treatment, the duration of anticoagulation treatment was used as a covariate while intubation was treated as a time-dependent variable. Among 2773 hospitalised patients with COVID-19, 786 (28%) received systemic therapeutic anticoagulation during their hospital course. This study was published as a letter to the editor.

**Ljitos** [13] performed a systematic assessment of venous thromboembolism (VTE) using complete duplex ultrasound in anticoagulated COVID-19 patients and described this in a ‘brief report’. The authors performed a retrospective study in two French ICUs where complete duplex ultrasound is performed as a standard of care. From 19 March to 11 April 2020, 26 consecutive patients with severe COVID-19 were screened for VTE. Seven patients were taking anticoagulants before hospital admission, 11 started anticoagulant use at hospital admission. Eight patients (31%) were treated with prophylactic anticoagulation, whereas 18 patients (69%) were treated with therapeutic anticoagulation. The overall rate of VTE in patients was 69%. All patients underwent mechanical ventilation, with prone positioning in 16 patients (62%). This study was published as a brief report.

The characteristics of the included studies are summarised in Tab. 1.

**Table 1:** Overview of the identified studies

Group	Author, year	Comparison	Start anticoagulants	Setting	Country	Study design	Outcomes	Publication type
A. Anticoagulant drug use before hospital admission	Tremblay, 2020 [8]	Anticoagulant use at moment of infection (therapeutic dose) vs patients not using anticoagulants at moment of infection	Pre-admission	Hospitalised and ambulatory COVID-19 patients	US	Retrospective, observational	Mortality, hospital admission, mechanical ventilation Propensity matched analysis	To the editor
A. Anticoagulant drug use before hospital admission	Klok, 2020 [2]	Use of long-term therapeutic anticoagulation vs prophylactic dose	Pre-admission	Patients with proven COVID-19 pneumonia admitted to the ICU	Netherlands	Observational	Mortality (HR), a composite outcome (symptomatic acute pulmonary embolism, deep vein thrombosis, ischaemic stroke, myocardial infarction and/or systemic arterial embolism) (corrected for competing risk of death)	Research paper update
A. Anticoagulant drug use before hospital admission	Russo, 2020 [9]	Pre-admission anticoagulant users vs non-users	Pre-admission	Emergency department	Italy	Retrospective, observational	Mortality Propensity score model	Research paper
A. Anticoagulant drug use before hospital admission	Sivaloganathan, 2020 [10]	Pre-admission antiplatelet/anticoagulant use vs non-users	Pre-admission	Hospitalised COVID-19 patients	UK	Retrospective, case control	Mortality (log rank, no correction for confounders) ICU admission	Correspondence
A. Anticoagulant drug use before hospital admission	Rossi, 2020 [11]	Chronic anticoagulant users vs non-users	Pre-admission	Elderly COVID-19 patients with coronary heart disease followed in the outpatient clinic	Italy	Retrospective observational	Mortality (corrected for age and gender)	Letter to the editor
B. Prophylactic anticoagulant drug use start at hospital admission	Tang, 2020 [7]	Prophylactic heparin vs non-users	Hospital admission	Hospitalised severe COVID-19 patients	China	Retrospective, observational	28-day mortality (adjusted for age, sex, underlying disease, platelet count, D‑dimer)	Research paper
C. Mixed or unclear start of anticoagulant	Paranjpe, 2020 [12]	Therapeutic dose vs (prophylactic dose or non-users)	Unclear	Hospitalised COVID-19 patients	US	Retrospective, observational	Invasive mechanical ventilation (no correction for confounders), mortality (no correction for confounders)	Letter
C. Mixed or unclear start of anticoagulant	Llitjos, 2020 [13]	Patients using a therapeutic dose of anticoagulant vs patients using a prophylactic dose of anticoagulant	7 patients pre-admission, 11 patients at admission	COVID-19 patients admitted to the ICU	France	Retrospective, observational	Mortality, pulmonary embolism, acute kidney injury, (comparison between 2 groups, no correction for confounders), renal replacement therapy, VTE (including non-symptomatic VTE)	Brief report

## Results

### Mortality

#### Group A: Anticoagulant drug use before hospital admission

**Klok** [2] studied 184 patients admitted to the ICU and compared patients on long-term therapeutic anticoagulation with patients who received pharmacological thromboprophylaxis. The use of long-term therapeutic anticoagulation was not associated with all-cause death (hazard ratio (HR) 0.79, 95% confidence interval (CI) 0.35–1.8).

**Tremblay** [8] performed two types of analysis for mortality, a time-to-event analysis and event analysis. Overall, 15.0% of the patients died. Of the patients receiving anticoagulation, 81 (33.6%) died, of the non-users 317 (11.1%) died. The time-to-event analysis (Kaplan-Meier) showed that there was no statistically significant difference in survival (*p* = 0.367). Event analysis also showed no difference in mortality between the two groups: HR 1.208, 95% CI 0.750–1.946.

**Russo** [9] studied 192 COVID-19 patients, 35 (18.5%) died during hospitalisation. Russo found no statistically significant difference (*p* = 0.678) between hospitalised COVID-19 patients on anticoagulant therapy with regard to survival (*n* = 20, 12.7%) or non-survival (*n* = 6, 17.1%). In a propensity score regression model, the unadjusted relative risk (RR) for the risk of death was 1.42, 95% CI 0.53–2.47, *p* = 0.493. In the adjusted model (adjusted for age, smoking, chronic obstructive pulmonary disease, hypertension, diabetes, coronary artery disease, heart failure, obesity, dyslipidaemia) the RR for the risk of death was 1.15, 95% CI 0.29–2.57, *p* = 0.995. Antithrombotic therapy before admission did not influence the clinical presentation of COVID-19 in terms in-hospital mortality.

**Sivaloganathan** [10] found that taking an anticoagulant agent before admission did not have a statistically significant effect on mortality in 31 patients with COVID-19 (*p* = 0.614) using the log-rank test, suggesting no protective effect. No correction for confounders was applied. However, the evident confounder in this analysis is the comorbidity of cardiovascular disease, itself an established risk factor for increased mortality in COVID-19 and thrombotic disorders.

**Rossi** [11] studied 70 elderly patients affected by COVID-19 interstitial pneumonia. All patients were affected by chronic heart disease. In a multivariate analysis (adjusted for age and male gender) Rossi found an adjusted HR of 0.38, 95% CI 0.17–0.58, *p* = 0.01 indicating that chronic use of direct oral anticoagulants is an independent parameter associated with a decreased mortality risk for this patient group.

#### Group B: Prophylactic anticoagulant drug use started at hospital admission

**Tang** [7] found no statistically significant difference on the 28-day mortality between prophylactic heparin users (*n* = 30, 30.3%) and non-users (*n* = 104, 29.7%) (*p* = 0.910) with severe COVID-19. The heparin treatment was associated with lower mortality in COVID-19 patients with a Sepsis-Induced Coagulopathy (SIC) score ≥4 (40.0% vs 64.2%, *p* = 0.029), but not in those with a SIC score <4 (29.0% vs 22.6%, *p* = 0.419). In a multivariate analysis (adjusted for age, sex ratio, underlying disease, prothrombin time, platelet count, D‑dimer) the adjusted odds ratio for mortality was 1.647 (95% CI 0.929–2.921, *p* = 0.088).

#### Group C: Mixed or unclear start of anticoagulant therapy

**Paranjpe** [12] studied 2773 hospitalised COVID-19 patients of which 786 received anticoagulants. In-hospital mortality for patients treated with anticoagulants was 22.5% (median survival 21 days). Of the patients who received a prophylactic dose or no anticoagulation, 22.8% died (median survival 14 days). In a multivariate proportional-hazards model (adjusted for age, sex, ethnicity, body mass index, history of hypertension, heart failure, atrial fibrillation, type 2 diabetes, anticoagulant use prior to hospitalisation and admission date), longer duration of anticoagulation treatment was associated with a reduced risk of mortality (adjusted HR of 0.86 per day, 95% CI 0.82–0.89, *p* < 0.001).

**Llitjos** [13] studied 26 patients admitted to the ICU and compared patients on a therapeutic dose of anticoagulants with patients who received a prophylactic dose or no anticoagulation. Three patients died, two of which received a therapeutic dose (11%) and one received a prophylactic dose (12%). The authors did not report whether this was a statistically significant difference.

### ICU admission

#### Group A: Anticoagulant drug use before hospital admission

**Sivaloganathan** [10] studied the relation between therapeutic anticoagulant use before hospital admission and ICU admission. Of the patients receiving therapeutic anticoagulant drugs, 5 (16.7%) required ICU admission, for the control group this was 7 (11.3%). The chi-square test showed this was not a statistically significant difference (*p* = 0.472). Correction for confounders was not applied.

#### Group B: Prophylactic anticoagulant drug use started at hospital admission

Not reported.

#### Group C: Mixed or unclear start of anticoagulant therapy

Not reported.

### Length of stay

#### Group A–C

Not reported.

### Thromboembolic complications

#### Group A: Anticoagulant drug use before hospital admission

**Klok** [2] developed a composite outcome of symptomatic acute pulmonary embolism, deep vein thrombosis, ischaemic stroke, myocardial infarction and/or systemic arterial embolism. The majority of thrombotic events were pulmonary embolisms (65/75, 87%). The crude cumulative incidence of the composite outcome was 57% (95% CI 47–67%), and after adjustment for competing risk of death 49% (95% CI 41–57%). The incidence rate was 13/patient-year (95% CI 6.1–27). In a competing risk model, chronic anticoagulation therapy at admission was associated with a lower risk of the composite outcome (HR 0.29, 95% CI 0.091–0.92).

#### Group B: Prophylactic anticoagulant drug use started at hospital admission

Not reported.

#### Group C: Mixed or unclear start of anticoagulant therapy

**Llitjos** [13] found that the overall rate of VTE was 69% of the 26 patients admitted to the ICU in their study. Pulmonary embolism was diagnosed in six patients (23%). The proportion of VTE events was significantly higher in patients treated with prophylactic anticoagulation when compared with the therapeutic anticoagulation group (100% vs 56%, respectively, *p* = 0.03). However, a high rate of thromboembolic events was found in COVID-19 patients treated with therapeutic anticoagulation, with 56% of venous thromboembolic events and six pulmonary embolisms.

### Ventilation

#### Group A: Anticoagulant drug use before hospital admission

**Tremblay** [8] performed two types of analysis for ventilation, a time-to-event analysis and event analysis. Overall, 13.8% of the patients required mechanical ventilation. The time-to-event analysis (Kaplan-Meier) showed that there was no statistically significant difference in ventilation (*p* = 0.742). The event analysis also showed no difference in ventilation between the two groups (HR 0.905, 95% CI 0.571–1.435).

#### Group B: Prophylactic anticoagulant drug use started at hospital admission

Not reported.

#### Group C: Mixed or unclear start of anticoagulant therapy

**Paranjpe** [12] found that patients who received therapeutic dose anticoagulants were more likely to require invasive mechanical ventilation (29.8% vs 8.1%, *p* < 0.001) as compared with those who received a prophylactic dose or did not receive anticoagulation.

**Llitjos** [13] reported that all included patients in both groups needed mechanical ventilation. However, all patients included in this study were admitted to the ICU.

### Acute kidney injury therapy

#### Group A–B

Not reported.

#### Group C: Mixed or unclear start of anticoagulant therapy

**Llitjos** [13] found that of the eight patients receiving prophylactic anticoagulation, 2 (25%) developed acute kidney injury. Of the 18 patients on therapeutic anticoagulation, 7 (39%) developed acute kidney injury. The authors did not report whether this was a statistically significant difference.

### Renal replacement therapy

#### Group A: Anticoagulant drug use before hospital admission

**Tremblay** [8] found that of the patients who underwent renal replacement therapy, 7 (2.9%) were on anticoagulant drugs versus 91 (3.2%) not on anticoagulant drugs or antiplatelet therapy (*p* = 0.051).

#### Group B: Prophylactic anticoagulant drug use started at hospital admission

Not reported.

#### Group C: Mixed or unclear start of anticoagulant therapy

**Llitjos** [13] found that of the patients who received a therapeutic dose of anticoagulation, 4 (22%) underwent renal replacement therapy. Of the patients on a prophylactic dose of anticoagulation, none (0%) underwent renal replacement therapy. The authors did not report whether this was a statistically significant difference.

## Level of evidence of the literature

The level of evidence was assessed according to the GRADE methodology (GRADE: Grading Recommendations Assessment, Development and Evaluation, http://www.gradeworkinggroup.org/). The level of evidence and risk of bias is shown in Tables S3 and S4 of the Electronic Supplementary Material.

### Mortality (crucial)

#### Group A: Anticoagulant drug use before hospital admission

Starting with a high level of evidence for observational studies (prognostic question), the level of evidence regarding the outcome of mortality was downgraded by two levels because of risk of bias (not all studies correct for confounders, one study had a very specific patient group, studies described in correspondence or letters to the editor so very little information on methodology, patient characteristics and outcomes) to ‘low’.

#### Group B: Prophylactic anticoagulant drug use started at hospital admission

Starting with a high level of evidence for observational studies (prognostic question), the level of evidence regarding the outcome of mortality was downgraded by one level because of risk of bias (patient characteristics of the two groups not described) and two levels because of imprecision (only one study available, small number of patients and very low number of events) to ‘very low’.

#### Group C: Mixed or unclear start of anticoagulant therapy

Starting with a high level of evidence for observational studies (prognostic question), the level of evidence regarding the outcome of mortality was downgraded by two levels because of risk of bias (patient groups not well described, follow-up not complete, one study described in a letter so very little information on patients, methods and results available), and one level because of imprecision (small number of patients and low number of events) to ‘very low’.

### ICU admission

#### Group A: Anticoagulant drug use before hospital admission

Starting with a high level of evidence for observational studies (prognostic question), the level of evidence regarding the outcome of ICU admission was downgraded by two levels because of risk of bias (no information on patient characteristics, no correction for important confounders) and one level because of imprecision (small study population) to ‘very low’.

### Length of stay

Not reported.

### Thromboembolic complications

#### Group A: Anticoagulant drug use before hospital admission

Starting with a high level of evidence for observational studies (prognostic question), the level of evidence regarding the outcome measure of thromboembolic complications was downgraded by one level because of risk of bias (absolute number of thromboembolic complication events unknown) and one level because of imprecision (low number of events) to ‘low’.

#### Group C: Mixed or unclear start of anticoagulant therapy

Starting with a high level of evidence for observational studies (prognostic question), the level of evidence regarding the outcome measure of thromboembolic complications was downgraded by one level because of risk of bias (no correction for confounders), one level for indirectness (the study only included severe COVID-19 patients admitted to the ICU) and one level for imprecision (very small sample size) to ‘very low’.

### Ventilation

#### Group A: Anticoagulant drug use before hospital admission

Starting with a high level of evidence for observational studies (prognostic question), the level of evidence regarding the outcome of ventilation was downgraded by one level because of risk of bias (no correction for confounders) and one level for imprecision (very few events) to ‘low’.

#### Group C: Mixed or unclear start of anticoagulant therapy

Starting with a high level of evidence for observational studies (prognostic question), the level of evidence regarding the outcome of ventilation was downgraded by one level because of risk of bias (no correction for confounders), one level because of imprecision (the two studies reported different results), and one level because of indirectness (specific patient group in one study) to ‘very low’.

### Acute kidney injury

#### Group C: Mixed or unclear start of anticoagulant therapy

Starting with a high level of evidence for observational studies (prognostic question), the level of evidence regarding the outcome of acute kidney injury was downgraded by one level because of risk of bias (no correction for confounders) and two levels because of imprecision (very small sample size of only one study) to ‘very low’.

### Renal replacement therapy

#### Group A: Anticoagulant drug use before hospital admission

Starting with a high level of evidence for observational studies (prognostic question), the level of evidence regarding the outcome of acute kidney injury was downgraded by one level because of risk of bias (no correction for confounders) and two levels because of imprecision (only one study available, low number of events) to ‘very low’.

#### Group C: Mixed or unclear start of anticoagulant therapy

Starting with a high level of evidence for observational studies (prognostic question), the level of evidence regarding the outcome of acute kidney injury was downgraded by one level because of risk of bias (no correction for confounders) and two levels because of imprecision (very small sample size of only one study) to ‘very low’.

## Conclusion

### Mortality

#### Group A: Anticoagulant drug use before hospital admission

The evidence suggests that anticoagulation therapy does not affect mortality. *Sources: Klok, Tremblay, Russo, Sivaloganathan, Rossi* **(low GRADE level)**.

#### Group B: Anticoagulant drug use started at hospital admission

Prophylactic anticoagulation therapy may have little to no effect on mortality but the evidence is very uncertain. *Source: Tang* **(very low GRADE level)**.

#### Group C: Mixed or unclear start of anticoagulant therapy

Anticoagulation therapy may have little to no effect on mortality but the evidence is very uncertain. *Sources: Llitjos, Paranjpe* **(very low GRADE level)**.

### ICU admission

Anticoagulation therapy (before hospital admission) may have little to no effect on ICU admission but the evidence is very uncertain. *Source: Sivaloganathan* **(very low GRADE level)**.

### Length of stay

Not reported.

### Thromboembolic complications

#### Group A: Anticoagulant drug use before hospital admission

Anticoagulant drug use may result in a slight reduction in thromboembolic complications. *Source: Klok* **(low GRADE level)**.

#### Group C: Mixed or unclear start of anticoagulant therapy

Anticoagulation therapy may reduce thromboembolic complications but the evidence is very uncertain. *Source: Llitjos* **(very low GRADE level)**.

### Ventilation

#### Group A: Anticoagulant drug use before hospital admission

Anticoagulation therapy may have little to no effect on ventilation but the evidence is very uncertain. *Source: Tremblay* **(very low GRADE level)**.

#### Group C: Mixed or unclear start of anticoagulant therapy

The evidence is very uncertain about the effect of anticoagulation therapy on ventilation. *Sources: Paranjpe, Llitjos* **(very low GRADE level)**.

### Acute kidney injury

#### Group C: Mixed or unclear start of anticoagulant therapy

The evidence is very uncertain about the effect of anticoagulation therapy on acute kidney injury. *Source: Llitjos* **(very low GRADE level)**.

### Renal replacement therapy

#### Group A: Anticoagulant drug use before hospital admission

The evidence is very uncertain about the effect of anticoagulation therapy on renal replacement therapy. *Source: Tremblay* **(very low GRADE level)**.

#### Group C: Mixed or unclear start of anticoagulant therapy

The evidence is very uncertain about the effect of anticoagulation therapy on renal replacement therapy. *Source: Llitjos* **(very low GRADE level)**.

Although it is clear that COVID-19 is associated with an increased prevalence of thromboembolic complications, there is insufficient evidence that therapeutic anticoagulation can improve outcome in these patients. None of the studies demonstrated improved mortality, except for one very small Italian study. Furthermore, none of the studies demonstrated a positive effect of anticoagulation on other outcome measures in COVID-19, such as ICU admission, length of hospital stay, thromboembolic complications, need for mechanical ventilation, acute kidney failure or need for renal replacement therapy, except for two studies demonstrating an association between anticoagulation and a lower incidence of pulmonary embolism. However, the level of evidence of all studies varied from ‘low’ to ‘very low’, according to the GRADE methodology.

## Discussion

This analysis of the literature, up until 17 July 2020, demonstrated that there was insufficient evidence to answer our objective on the effect of anticoagulation on outcome in COVID-19 patients, especially due to the low scientific quality of the described studies. Multiple randomised controlled trials examining therapeutic anticoagulation in COVID-19 are expected to present their results in 2021. Once these become available, they can be added to this review of the literature.

### CAPACITY registry

The same research question was studied in the CAPACITY registry, an international initiative to evaluate the role of cardiovascular disease in patients hospitalised with COVID-19 [14]. In August 2020, 61 hospitals from 13 countries contributed to the data collection and 40% of the hospitalised Dutch patients were entered into the database. In this registry, 694 out of 4921 hospitalised COVID-19 patients received either vitamin K antagonists or direct oral anticoagulants (OAC) because of pre-existing conditions before admission. OAC patients were older and more often had comorbidities than patients without OAC. Cardiovascular risk factors, such as hypertension and diabetes mellitus, pre-existing cardiac disease, such as atrial fibrillation and heart failure, and chronic obstructive pulmonary disease were more prevalent in the OAC patients. The primary outcome of in-hospital mortality was higher among patients receiving OAC than those without OAC (HR 1.83, 95% CI 1.57–2.12). However, after multivariable Cox regression analysis (adjusting for age, chronic obstructive pulmonary disease, hypertension and heart failure) the hazard ratio was 1.05 (95% CI 0.90–1.23), indicating no difference in mortality, similar to the studies described in the literature search. We did, however, find an association between anticoagulation use before admission and a 67% lower incidence of pulmonary embolism. The CAPACITY study is currently under peer-review. If there is an update of the literature search on the topic of anticoagulation in the future, this recommendation will be updated accordingly.

### Recommendations

Based on the present literature search and the results from the CAPACITY registry, there is no reason to change the current recommendations on anticoagulation in COVID-19 patients (Leidraad COVID-19 Coagulopathie: https://www.demedischspecialist.nl/sites/default/files/Leidraad%20COVID-19%20coagulopathie.pdf). It is expected that results from randomised controlled trials will soon become available. Until then, the recommendations remain unchanged (See Box 1).

#### Box 1 Recommendations

- Administer prophylactic dose anticoagulation to all patients admitted with COVID-19.
- Administer double standard prophylactic dose anticoagulation to COVID-19 patients admitted to the ICU.
- There is no indication for therapeutic dose anticoagulation in COVID-19, in the absence of pre-existing indications.
- When convincing evidence becomes available to change these recommendations, the multidisciplinary guideline will be updated. (https://richtlijnendatabase.nl/richtlijn/antitrombotisch_beleid/antitrombotisch_beleid_-_korte_beschrijving.html).

### Gaps in evidence

The initial research question remains unanswered: Should patients with COVID-19 be treated with prophylactic or therapeutic dose anticoagulation to prevent cardiovascular and thromboembolic complications and improve clinical outcome?

## Supplementary Information

Table S1 Literature search strategy

Table S2 Excluded studies

Table S3 Evidence for intervention studies. Evidence table for intervention studies (randomised controlled trials and non-randomised *observational* studies [cohort studies, case-control studies, case series])

Table S4 Risk of bias for intervention studies (observational: non-randomized clinical trials, cohort and case-control studies)
