# Supplementary material for: Effect of anticoagulant therapy in COVID-19 patients
Source: Neth Heart J. 2021 Apr 16;29(Suppl 1):35–44. doi: 10.1007/s12471-021-01574-7 (PMC8050812; doi:10.1007/s12471-021-01574-7)
Supplement: Supplementary file 1 — Table S1 Literature search strategy [file 12471_2021_1574_MOESM1_ESM.docx]

**Table S1**. Literature search strategy

OVID/Medline

1 ((exp Coronavirus/ or Coronavirus Infections/ or pneumonia virus*.ti,ab,kf. or cov.ti,ab,kf.) and ((outbreak or wuhan).ti,ab,kf. or novel.af. or '19'.ti,ab,kf. or '2019'.ti,ab,kf. or epidem*.af. or epidemy.af. or epidemic*.af. or pandem*.af. or new.ti,ab,kf.)) or (coronavirus* or 'corona virus*' or ncov or '2019ncov' or 'covid19' or "covid 19" or "sars cov 2" or 'sars2' or "ncov 2019" or "sars coronavirus 2" or "sars corona virus 2" or "severe acute respiratory syndrome cov 2" or "severe acute respiratory syndrome cov2" or "severe acute respiratory syndrome cov*").ti,ab,kf. (46908)

2 limit 1 to dt="20191201-20220101" (34250)

5 letter/ (1089279)

7 (anti coagulant* or anticoagulant* or anticoagulat* or anit coagulat* or antivitamin k or vitamin k antagonist* or choay or depolymerized heparin or low molecular heparin or low molecular weight heparin or traxyparine or unfractionated heparin).ti. (37017)

8 2 and 7 (24)

9 from 8 keep 1-24 (24)

Embase

| No. | Query | Results |
| --- | --- | --- |
| #15 | #4 AND #13 AND #14 | 8 |
| #14 | 'anti coagulant*':ti OR 'anticoagulant*':ti OR 'antithrombotic*':ti OR anticoagulat*:ti | 52776 |
| #13 | 'letter'/it | 1108495 |
| #4 | ('coronavirus disease 2019'/exp OR (('coronavirinae'/exp OR 'coronavirus infection'/de OR coronavirus*:ti,ab,kw OR 'corona virus*':ti,ab,kw OR 'pneumonia virus*':ti,ab,kw OR cov:ti,ab,kw OR ncov:ti,ab,kw) AND (outbreak:ti,ab,kw OR wuhan:ti,ab,kw)) OR covid19:ti,ab,kw OR 'covid 19':ti,ab,kw OR ((coronavirus*:ti,ab,kw OR 'corona virus*':ti,ab,kw) AND 2019:ti,ab,kw) OR 'sars cov 2':ti,ab,kw OR sars2:ti,ab,kw OR 'coronavirus*':ti,ab,kw OR 'corona virus*':ti,ab,kw OR 'ncov 2019':ti,ab,kw OR ncov:ti,ab,kw OR 'sars coronavirus 2':ti,ab,kw OR 'sars corona virus 2':ti,ab,kw OR 'severe acute respiratory syndrome cov 2':ti,ab,kw OR 'severe acute respiratory syndrome cov2':ti,ab,kw) AND [2019-2020]/py | 25663 |
