# Supplementary material for: Effect of anticoagulant therapy in COVID-19 patients
Source: Neth Heart J. 2021 Apr 16;29(Suppl 1):35–44. doi: 10.1007/s12471-021-01574-7 (PMC8050812; doi:10.1007/s12471-021-01574-7)
Supplement: Supplementary file 2 — Table S2 Excluded studies [file 12471_2021_1574_MOESM2_ESM.docx]

**Table S2.** Excluded studies

| Author and year | Reason for exclusion |
| --- | --- |
| Maldonado (2020) | No comparison between patients on anticoagulants and not on anticoagulants. There are 3 studies included in a qualitative analysis (1 is included in our set, 1 has no comparison, 1 is case study of 1 or 2 patients) |
| Fauvel (2020) | Wrong I/C: study compares patients with pulmonary embolism with patients without pulmonary embolism |
| Tremblay (2020) | Same as rayyan-90706111 |
| Al-Samkari (2020) | Wrong C: the study compares coagulation and inflammatory parameters. Not the use of anticoagulants : Coagulation and inflammatory parameters were compared between patients with and without coagulation-associated complications. Exclude want parameters (niet use zelf) vgl met complications |
| Cummings (2020) | No comparison between patients on anticoagulants and not on anticoagulants |
| Helms (2020) | Wrong C: the study compares covid and non covid patients on Thrombotic and ischemic complications |
| Liu (2020) | Proof-of-concept trial |
| Viecca (2020) | Proof-of-concept study, wrong I (antiplatelet), wrong O (hypoxemia) |
| Lodigiani (2020) | Study does not compare patients on anticoagulants with patients not on anticoagulants but thromboembolytic events in patients admitted to IC and to ward |
| Porfidia (2020) | Does not include original data, is a comment on the paper of Tang |
| Aghamohammadi (2020) | No original data |
| Rossi (2020) | Same as rayyan-90706105 |
| Pierce-Williams (2020) | Pregnant women, wrong outcome (severe vs critical) |
| Somani (2020) | Pre-print, not peer reviewed |
| Trigonis (2020) | Wrong C: all patients were using prophylactic anticoagulants so wrong comparison |
| Zeng (2020) | Wrong I/C: high Padua prediction score vs low PPS |
| Secco (2020) | Wrong C: death vs survivor instead of |
| Aghamohammadi (2020) | same as rayyan-88799905 |
| Coto-Hernández (2020) | No original data, comment on the paper of Tang |
| Doganci (2020) | No original data |
| Kow (2020) | No original data |
| Menezes-Rodrigues (2020) | No original data |
| Sivaloganathan (2020) | Same as rayyan-90528100 |
| Ayerbe (2020) | No information on dosage and start moment of anticoagulant drugs |
