# Supplementary material for: Effect of anticoagulant therapy in COVID-19 patients
Source: Neth Heart J. 2021 Apr 16;29(Suppl 1):35–44. doi: 10.1007/s12471-021-01574-7 (PMC8050812; doi:10.1007/s12471-021-01574-7)
Supplement: Supplementary file 3 — Table S3 Evidence for intervention studies. Evidence table for intervention studies (randomised controlled trials and non-randomised observational studies [cohort studies, case-control studies, case series]) [file 12471_2021_1574_MOESM3_ESM.docx]

**Table S3 Evidence for intervention studies**

**Evidence table for intervention studies (randomized controlled trials and non-randomized *observational* studies [cohort studies, case-control studies, case series])^1^**

*This table is also suitable for diagnostic studies (screening studies) that compare the effectiveness of two or more tests. This only applies if the test is included as part of a test-and-treat strategy – otherwise the evidence table for studies of diagnostic test accuracy should be used.*

| **Study reference** | **Study characteristics** | **Patient characteristics ^2^** | **Intervention (I)** | **Comparison / control (C) ^3^** | **Follow-up** | **Outcome measures and effect size ^4^** | **Comments** |
| --- | --- | --- | --- | --- | --- | --- | --- |
| Paranjpe, 2020 | Type of study: retrospective observational  Setting and country: hospitalized patients with COVID-19, US  Funding and conflicts of interest: study supported by U54 TR001433-05, National Center  for Advancing Translational Sciences, National Institutes of Health. Some of the authors received funding from (pharmaceutical) companies, NA | Inclusion criteria: NA  Exclusion criteria: N  N total at baseline: 2773  Intervention: 786  Control: 1987  Important prognostic factors^2^:  *NA*  Groups comparable at baseline?  There is no information available on patient characteristics in both groups | Median time from admission to AC initiation  was 2 days (IQR: 0 to 5 days). Median duration of AC treatment was 3 days (IQR: 2 to 7 days). | Prophylactic dose AC or no AC | Length of follow-up:  NA  Loss-to-follow-up:  NA  Incomplete outcome data:  NA | *1. Mortality*  In-hospital  mortality for patients treated with AC: 22.5%, median survival 21 days  No treatment dose: 22.8%, median survival 14 days  Multivariate proportional  hazards model (adjusted for age, sex, ethnicity, body mass index, history of hypertension,  heart failure, atrial fibrillation, type 2 diabetes, AC use prior to hospitalization, and admission date) , longer duration of AC treatment was associated with a reduced risk of mortality (adjusted HR of 0.86 per day; 95% confidence interval: 0.82 to  0.89; p < 0.001).  *2. IC admission*  Not reported  *3. Length of stay*  Not reported  *4. Thromboembolic complications*  Not reported  *5. Ventilation*  Defined as invasive mechanical ventilation.  Patients  who received treatment-dose AC were more likely to  require invasive mechanical ventilation (29.8% vs 8.1%; p < 0.001)  *6. Acute kidney injury* Not reported  *7. Renal replacement*  *Therapy*  Not reported | 1. Mortality,  2. IC-admission,  3. length of stay,  4. thromboembolic complications (pulmonary embolism, stroke, transient ischemic attack),  5. ventilation,  6. acute kidney injury(AKI) (volgens KDIGO criteria),  7. use of renal replacement therapy |
| Tremblay, 2020 | Type of study: retrospective observational  Setting and country: hospitalized patients with COVID-19, US  Funding and conflicts of interest: NA, NA | Inclusion criteria:  consecutive patients with laboratory confirmed  COVID-19 between 1 March 2020 and 1 April 2020  Exclusion criteria:  younger than 18 years of age and/or insufficient clinical  documentation because they had been diagnosed at a rapid  testing center  N total at baseline: 3772  Intervention: 241  Control: 2859  Propensity  matching yielded 139 patients who received AC and 417 patients who did not receive treatment  Important prognostic factors^2^:  *Age, mean (SD)*  *I: 73.25 (13.6) year*  *C: 52.36 (17.6) year*  Sex (N, % males)  I: 133 (55.2)  C: 1533 (53.6)  Groups comparable at baseline?  Yes  After propensity matching | Patients who were on AC prior to COVID-19 infection | Patients who were not on AC or antiplatelet therapy | Length of follow-up: not reported  Loss-to-follow-up: not reported  Incomplete outcome data: not reported | *1. Mortality*  HR 1.208 (95% CI, 0.750-1.946),  *2. IC admission*  Not reported  *3. Length of stay*  Not reported  *4. Thromboembolic complications*  Defined as overt thrombosis  I: 3 (1.2%)  C: 29 (1.0%)  P=0.076  Defined as major bleeding  I: 3 (1.2%)  C: 11 (0.4%)  P=0.007  *5. Ventilation*  Defined as Intubation-mechanical  Ventilation  HR 0.905 (95% CI, 0.571-1.435),  *6. Acute kidney injury* Not reported  *7. Renal replacement*  *Therapy*  Defined as new RRT (of total group)  I: 7 (2.9%)  C: 91 (3.2%)  P=0.051 |  |
| Klok, 2020 | Type of study: Retrospective observational  Setting and country: COVID-19 patients admitted to the ICUs, Netherlands  Funding and conflicts of interest: funding outside the submitted work, no conflicts of interest declared | Inclusion criteria:  Exclusion criteria:  N total at baseline: 184  Intervention: 17  Control: 167  Important prognostic factors^2^:  *NA*  Groups comparable at baseline?  NA | Patients on long-term therapeutic anticoagulation for various reasons,  continued at ICU admission | Pharmacological thromboprophylaxis  according to local hospital protocols | Length of follow-up:  Patients  were censored upon ICU discharge, when they died, or at April 22nd  2020, whichever came first.  Loss-to-follow-up:  NA  Incomplete outcome data:  NA | *1. Mortality*  Use of long-term therapeutic  anticoagulation was not associated with all-cause death (HR 0.79, 95%CI 0.35–1.8).  *2. IC admission*  Not reported  *3. Length of stay*  Not reported  *4. Thromboembolic complications*  In the competing risk model, the hazard ratio (HR) for the  composite outcome (of symptomatic acute pulmonary embolism (PE), deep-vein thrombosis, ischemic stroke, myocardial infarction and/or systemic arterial embolism) associated with long-term therapeutic anticoagulation  was 0.29 (95%CI 0.091–0.92).  *5. Ventilation*  Not reported  *6. Acute kidney injury* Not reported  *7. Renal replacement*  *Therapy*  Not reported |  |
| Llitjos, 2020 | Type of study: retrospective observational  Setting and country: COVID-19 patients admitted to the ICU  Funding and conflicts of interest: NA, no conflicts of interest declared. | Inclusion criteria: NA  Exclusion criteria: NA  N total at baseline: 26  Intervention: 8  Control: 18  Important prognostic factors^2^ (total group):  *Age*  *I: 67.5 (53.5-76.2)*  *C: 68 (45-72.7)*  *Sex (male:*  *I: 14 (78%)*  *C: 6 (75%)*  Groups comparable at baseline?  Yes, based on age, sex and chronic medical condition | Therapeutic anticoagulation  Dose: left to the discretion of the treating physician. Patients treated with  therapeutic anticoagulation received either low molecular weight  heparin or unfractionated heparin with anti-Xa monitoring, with  therapeutic levels of 0.3 to 0.7 U/mL of anti-Xa activity | Prophylactic anticoagulation | Length of follow-up:  March 19 to April 11, 2020  Loss-to-follow-up:  16 patients were discharged  from the ICU, and seven continued to receive mechanical ventilation  Incomplete outcome data:  Intervention: 0  Control: 0 | *1. Mortality*  I: 2 (11%)  C: 1 (12%)  NS, no P value reported  *2. IC admission*  Not reported  *3. Length of stay*  Not reported  *4. Thromboembolic complications*  Defined as pulmonary embolism  I:6 (33%)  C: 0 (0%)  NS, no P value reported  *5. Ventilation*  Defined as mechanical ventilation *I: 18 (100%) C: 8 (100%)* NS, no P value reported  *6. Acute kidney injury*  I:7 (39%)  C:2 (25%)  NS, no P value reported  *7. Renal replacement*  *Therapy*  Defined as use of renal replacement therapy  I: 4 (22%) C: 0 (0%) NS, no P value reported |  |
| Tang, 2020 | Type of study: Retrospective observational  Setting and country: Patients classified as having severe COVID-19 in Tongji hospital, China  Funding and conflicts of interest: National Mega Project on Major infectious Disease Prevention of  China, no conflicts of interest declared | Inclusion criteria:  Consecutive patients with severe COVID-19 admitted to Tongji Hospital of Huazhong University of Science and Technology in Wuhan from  January 1 to February 13, 2020  Exclusion criteria: bleeding diathesis, hospital stay < 7 days, lack of information  about coagulation parameters and medications, and age < 18 years  N total at baseline: 449  Intervention: 99  Control: 350  Important prognostic factors^2^: The paper compares survivors vs nonsurvivors  Total age: 65.1 ± 12.0  Sex ratio male/female: 268/181  Underlying disease: 272 (60.6%)  Groups comparable at baseline?  Not able to assess because the paper compares survivors with nonsurvivors | Patients receiving unfractionated heparin or low molecular weight heparin (LMWH) for 7 days or longer  N = 94 received LMWH  (40-60 mg enoxaparin/d) and five received unfractionated heparin  (10 000-15 000 U/d), no anticoagulants other than heparin had been used for 7 days or longer in our patients | Patients not receiving heparin (not further defined) | Length of follow-up:  28 days  Loss-to-follow-up:  0  Incomplete outcome data: NA | *1. Mortality*  28 day mortality  I: N= 30  C: N=104 No difference on the 28-day mortality was found between heparin users and nonusers (30.3% vs 29.7%,  P = .910).  The heparin treatment was associated with lower mortality in patients with sepsis-induced coagulopathy SIC score ≥ 4 (40.0% vs 64.2%, P = .029), but not in those with SIC score < 4 (29.0% vs 22.6%, P = .419).  Multivariate analysis (adjusted for age, sex ratio, underlying disease, prothrombin time, platelet count, D-dimer)  aOR 1.647 (95%CI 0.929-2.921) P=.088  *2. IC admission*  Not reported  *3. Length of stay*  Not reported  *4. Thromboembolic complications*  Not reported  *5. Ventilation*  Not reported  *6. Acute kidney injury* Not reported  *7. Renal replacement*  *Therapy*  Not reported |  |
| Russo, 2020 | Type of study: Retrospective observational  Setting and country: COVID-19 patients admitted to emergency department of five Italian hospitals, Italy  Funding and conflicts of interest: None, none declared | Inclusion criteria: confirmed COVID-19 patients patients admitted from February 2020 to April 2020 for fever and dyspnea to Emergency Department (ED) of five  Italian Hospitals (Humanitas Hospital of Milan, Fatebenefratelli Hospital of  Naples, Bergamo Hospital, Rivoli Hospital of Turin, Health Authority  Bergamo East)  Exclusion criteria: Discontinuation of antithrombotic  therapy during hospitalization  N total at baseline: 192  Intervention: 26  Control: 166  Important prognostic factors^2^:  *Age*  *I: 77.81±9.46 vs ;*  *C:66.07±15.35*  *P<0.001*  *Sex*  *I: NA*  *C: NA*  Groups comparable at baseline?  No. Patients on anticoagulants had older age, higher prevalence of hypertension (80.8 % vs 54.2 %; P=0.02), atrial fibrillation (84.6 % vs 1.2 %; P<0.001), heart failure (30.8 % vs 7.2 %; P = 0.001), CKD (19.2 % vs 1.2 %; P = 0.012), previous stroke (23.1 % vs 6.0 %; P = 0.011) and CAD (30.8 % vs 10.8 %; P= 0.009) | 18 (9.4 %) were taking non-vitamin K oral anticoagulant (NOAC) and 8 (4.2 %) patients were on well-controlled vitamin K  oral anticoagulant (VKA) before admission | Patients not using anticoagulants before admission | Length of follow-up:  patients admitted from February 2020 to April 2020  Loss-to-follow-up: NA  Incomplete outcome data: It is unclear when the analysis was performed and how many patients were still hospitalized at the time of the analysis | *1. Mortality*  I: 20 (12.7%)  C: 6 (17.1%)  P=0.678  Propensity score regression model  Unadjusted  RR 1.42 (95%CI 0.53 – 2.47) P=0.493  Adjusted (age, smoke, chronic obstructive pulmonary disease (COPD), hypertension, diabetes, coronary artery disease (CAD), heart failure, obesity, dyslipidemia, stroke, and  chronic kidney disease (CKD))  aRR 1.15 (95%CI 0.29 – 2.57) P=0.995  *2. IC admission*  Not reported  *3. Length of stay*  Not reported  *4. Thromboembolic complications*  Not reported  *5. Ventilation*  Not reported  *6. Acute kidney injury* Not reported  *7. Renal replacement*  *Therapy*  Not reported |  |
| Sivaloganathan, 2020 | Type of study: Case control propensity matched  Setting and country: admitted COVID-19 patients, UK  Funding and conflicts of interest: funded by the National Institute for Health Research (NIHR), no conflicts of interest declared | Inclusion criteria: patients while admitted as an inpatient in Brighton and Sussex  University Hospitals NHS Trust between the 7 March and 9  April 2020  Exclusion criteria:  N total at baseline:  Intervention: 31  Control: 62  Important prognostic factors^2^:  *Age (mean)*  *I: 80.5 y*  *C: 80.2 y*  Groups comparable at baseline?  Controls were selected from the study population with a  limited propensity matching by age and sex to two controls who were not taking the medication of interest using a ‘nearest  neighbour’ method.  There was no information available on other prognostic factors. | Patients who took anticoagulants before admission, of which 23% (n = 7) were on warfarin, 39%  (n = 12) apixaban, 3% (n = 1) dabigatran, 6% LMWH  (n = 2), and 29% (n = 9) rivaroxaban | Controls were selected from the study population with a  limited propensity matching by age and sex to two controls who were not taking the medication of interest using a ‘nearest  neighbour’ method. | Length of follow-up:  Patients admitted between the 7 March and 9  April 2020.  Mortality followed up to 11 May 2020  Loss-to-follow-up:  NA  Incomplete outcome data:  NA | *1. Mortality*  *Log rank test*  Being on an anticoagulant  agent before admission did not have a statistically  significant effect on mortality in patients with COVID-  19 (P = 0.614)  *2. IC admission*  I: 16.7%  C: 11.3%  Chi-square: P=0.472  There was no statistically significant difference in the percentage of patients admitted to the intensive care unit  *3. Length of stay*  Not reported  *4. Thromboembolic complications*  Not reported  *5. Ventilation*  Not reported  *6. Acute kidney injury* Not reported  *7. Renal replacement*  *Therapy*  Not reported |  |
| Rossi, 2020 | Type of study: Retrospective observational  Setting and country: elderly COVID-19 patients with chronic heart disease, Italy  Funding and conflicts of interest: NA, no conflicts of interest declared | Inclusion criteria: elderly patients affected by COVID-19 interstitial pneumonia between February 25, 2020, and April 20, 2020.  All the patients had chronic heart disease and were followed in the divisional outpatient clinic of the Cardiology Unit  of the Policlinico of Modena Hospital.  Exclusion criteria: NA  N total at baseline: 70  Intervention: 26  Control: 44  Important prognostic factors^2^:  *Age (median total group):*  79 years; range: 70–92)  Groups comparable at baseline? NA | Chronic intake of anticoagulants  26/70 patients (37.1%) were treated with direct oral  anticoagulants (DOAC) which underlying indication was pulmonary embolism (n = 7; 26.9%), deep vein thrombosis (n = 6; 23%) or atrial fibrillation (n = 13; 50%).  The majority of patients received rivaroxaban  (n = 11; 42.3%); followed by apixaban (n = 9; 34.6%),  edoxaban (n = 4; 15.4%), and dabigatran (n = 2; 7.7%). | Elderly COVID-19 patients with chronic heart disease not on chronic intake of anticoagulants | Length of follow-up:  patients affected by COVID-19  interstitial pneumonia between February 25, 2020, and April 20, 2020  The follow-up ended on May 5, 2020  Loss-to-follow-up: NA  Incomplete outcome data: NA | *1. Mortality*  Log-Rank (Mantel-Cox) = 9.767  P=0.01  Multivariate analysis (adjusted for age and male gender) aHR 0.38; 95%CI 0.17 – 0.58; P=0.01  Chronic DOAC intake is an independent parameter associated with a decreased mortality risk  *2. IC admission*  Not reported  *3. Length of stay*  Not reported  *4. Thromboembolic complications*  Not reported  *5. Ventilation*  Not reported  *6. Acute kidney injury* Not reported  *7. Renal replacement*  *Therapy*  Not reported |  |

**Notes:**

1. **Prognostic balance between treatment groups is usually guaranteed in randomized studies, but non-randomized (observational) studies require matching of patients between treatment groups (case-control studies) or multivariate adjustment for prognostic factors (confounders) (cohort studies); the evidence table should contain sufficient details on these procedures**
2. **Provide data per treatment group on the most important prognostic factors [(potential) confounders]**
3. **For case-control studies, provide sufficient detail on the procedure used to match cases and controls**
4. **For cohort studies, provide sufficient detail on the (multivariate) analyses used to adjust for (potential) confounders**
