# Supplementary material for: Effect of anticoagulant therapy in COVID-19 patients
Source: Neth Heart J. 2021 Apr 16;29(Suppl 1):35–44. doi: 10.1007/s12471-021-01574-7 (PMC8050812; doi:10.1007/s12471-021-01574-7)
Supplement: Supplementary file 4 — Table S4 Risk of bias for intervention studies (observational: non-randomized clinical trials, cohort and case-control studies) [file 12471_2021_1574_MOESM4_ESM.docx]

Table S4 **Risk of bias for intervention studies (observational: non-randomised clinical trials, cohort and case-control studies)**

| **Study reference**  (first author, year of publication) | **Bias due to a non-representative or ill-defined sample of patients?**^1^  (unlikely/likely/unclear) | **Bias due to insufficiently long, or incomplete follow-up, or differences in follow-up between treatment groups?^2^**  (unlikely/likely/unclear) | **Bias due to ill-defined or inadequately measured outcome ?^3^**  (unlikely/likely/unclear) | **Bias due to inadequate adjustment for all important prognostic factors?^4^**  (unlikely/likely/unclear) |
| --- | --- | --- | --- | --- |
| Paranjpe 2020 | Likely (Characteristics of groups are not described) | Unclear (no information available) | Unlikely | Mortality (unlikely)  Ventilation : likely (no correction for confounders) |
| Llitjos 2020 | Unlikely | Likely (for mortality, some patients are still hospitalized at the time of the analysis) | Unlikely | Likely (no correction for confounders but groups seemed comparable) |
| Klok 2020 | Unlikely | Unclear (for mortality, some patients still at the ic) | Unlikely | Unlikely |
| Tremblay 2020 | Unclear (unclear if patients received prophylactic AC in the hospital, the no of patients receiving it not mentioned) | Unclear (information NA) | Unlikely | Mortality: unlikely  Ventilation: likely (no correction for confounders)  Renal replacement therapy: likely (no correction for confounders) |
| Tang, 2020 | Unclear (comparison in the study is between survivors and nonsurvivors) | unlikely | unlikely | Unlikely |
| Russo, 2020 | Unlikely | unlikely | unlikely | Unlikely (propensity score matched) |
| Sivaloganathan, 2020 | Likely (there is no information on the patient characteristics except age) | unlikely | unlikely | Likely ( propensity score matching was applied but only for age and gender) |
| Rossi, 2020 | Likely (elderly COVID-19 patients with chronic heart disease) | Unclear (no information available) | Unlikely | Likely (only adjusted for age and male gender) |

1. **Failure to develop and apply appropriate eligibility criteria: a) case-control study: under- or over-matching in case-control studies; b) cohort study: selection of exposed and unexposed from different populations.**
2. **2 Bias is likely if: the percentage of patients lost to follow-up is large; or differs between treatment groups; or the reasons for loss to follow-up differ between treatment groups; or length of follow-up differs between treatment groups or is too short. The risk of bias is unclear if: the number of patients lost to follow-up; or the reasons why, are not reported.**
3. **Flawed measurement, or differences in measurement of outcome in treatment and control group; bias may also result from a lack of blinding of those assessing outcomes (detection or information bias). If a study has hard (objective) outcome measures, like death, blinding of outcome assessment is not necessary. If a study has “soft” (subjective) outcome measures, like the assessment of an X-ray, blinding of outcome assessment is necessary.**
4. **Failure to adequately measure all known prognostic factors and/or failure to adequately adjust for these factors in multivariate statistical analysis.**
